# ANXA2 is correlated with the molecular features and clinical prognosis of glioma, and acts as a potential marker of immunosuppression

**DOI:** 10.1038/s41598-021-00366-8

**Published:** 2021-10-21

**Authors:** Kaiming Ma, Xin Chen, Weihai Liu, Yang Yang, Suhua Chen, Jianjun Sun, Changcheng Ma, Tao Wang, Jun Yang

**Affiliations:** 1 Department of Neurosurgery, Peking University Third Hospital, 49 North Garden Rd, Haidian District, Beijing, 100191 China; 2 Center for Precision Neurosurgery and Oncology of Peking University Health Science Center, Beijing, China

**Keywords:** Cancer, Surgical oncology, Oncology, Diseases, Cancer, Cancer therapy, CNS cancer, Tumour biomarkers, Biomarkers, Biomarkers, Prognostic markers

## Abstract

Recent studies have shown that ANXA2 is important in the development of many cancers, while its role in glioma-related immune response remains unclear. We aimed to comprehensively investigate its biological characteristics and clinical value in glioma. We analyzed 699 glioma samples from The Cancer Genome Atlas as training cohort and 325 samples from the Chinese Glioma Genome Atlas as validation cohort. All the statistical analyses and figures were generated with R. ANXA2 was overexpressed significantly in high-grade glioma, isocitrate dehydrogenase wild-type and mesenchymal-subtype glioma. ANXA2 was a special indicator of mesenchymal subtype. The survival analysis showed that highly-expressed ANXA2 was related to worse survival status as an independent factor of poor prognosis. Further gene ontology analysis showed that ANXA2 was mainly involved in immune response and inflammatory activities of glioma. Subsequent correlation analysis showed that ANXA2 was positively correlated with HCK, LCK, MHC II, STAT1 and interferon but negatively with IgG. Meanwhile, ANXA2 was positively related to the infiltration of tumor-related macrophages, regulatory T cells and myeloid-derived suppressor cells. Our study revealed that ANXA2 is a biomarker closely related to the malignant phenotype and poor prognosis of glioma, and plays an important role in immune response, inflammatory activity and immunosuppression.

## Introduction

Glioma is the most common and fatal primary tumor of the central nervous system (CNS), accounting for 80.8% of CNS primary malignant tumors and 88.10% of deaths due to CNS tumors^1^. Malignant CNS tumors, represented by gliomas, are now the third most common cause of cancer-related death in people over 40 years of age^1^. Standard treatment for glioma now includes surgical excision, chemotherapy and radiation therapy^2, 3^, but this is insufficient to combat cancer progression^2^. The molecular-targeted therapeutic drug bevacizumab and tumor treating fields have also been approved by the Food and Drug Administration for the treatment of glioblastoma (GBM)^3, 4^. Immunotherapy has been used gradually in the clinic in recent years, but the overall therapeutic response of glioma is poor, especially GBM^3, 4^. Therefore, there is an urgent need to find more relevant glioma biomarkers that can act as therapeutic targets.

ANXA2 is an important member of the annexin family of proteins expressed on the surface of endothelial cells, macrophages, mononuclear cells and various types of cancer cells^5^. Free cytoplasmic ANXA2 exists as a 36-kDa protein^6^. There are four forms of ANXA2, secretory, membrane-bound, cytoplasmic, and nuclear^7^. The ANXA2 protein can exist as a monomer, heterodimer, or heterotetramer in vivo^6^. ANXA2 is involved in a variety of cellular functions, including vesicle transport, cell division, calcium signaling and cell growth^8^. Many functions of AN XA2 are regulated by a variety of posttranslational modifications^8^. In recent years, ANXA2 has been shown to play an important role in several cancers^5, 9^, and it promotes numerous processes associated with tumor progression, such as tumor proliferation, migration, epithelial mesenchymal transformation (EMT), invasion, stem cell formation, and resistance to therapy, such as radiotherapy, chemotherapy and immunotherapy^10^. AXNA2 was proved to be correlated with glioma grade and an unfavorable prognosis of GBM via the transcriptome profiling data of The Cancer Genome Atlas (TCGA)^11^, which greatly expands the previous analysis.

This makes ANXA2 a potential candidate target for tumor immunotherapy in the future. However, previous studies of ANXA2 in glioma have mostly focused on GBM, and most of them were in vitro studies. There are few comprehensive reports on the role of ANXA2 in glioma-related immune response based on large samples, which limits the development of clinically viable ANXA2-related therapies.

Therefore, we used RNA sequencing (RNA-seq) data from TCGA as training cohort and then validated our findings in the Chinese Glioma Genome Atlas (CGGA), analyzing a total of 1024 glioma cases. We aimed to reveal the role of ANXA2 in the malignant behavior of glioma comprehensively through conducting an analysis with large samples. This study provides a powerful theoretical basis for the design of ANXA2-targeted therapeutics for glioma.

## Results

### ANXA2 is significantly highly-expressed in high-grade glioma (HGG) and isocitrate dehydrogenase (IDH) wild-type glioma

**Figure 1:**
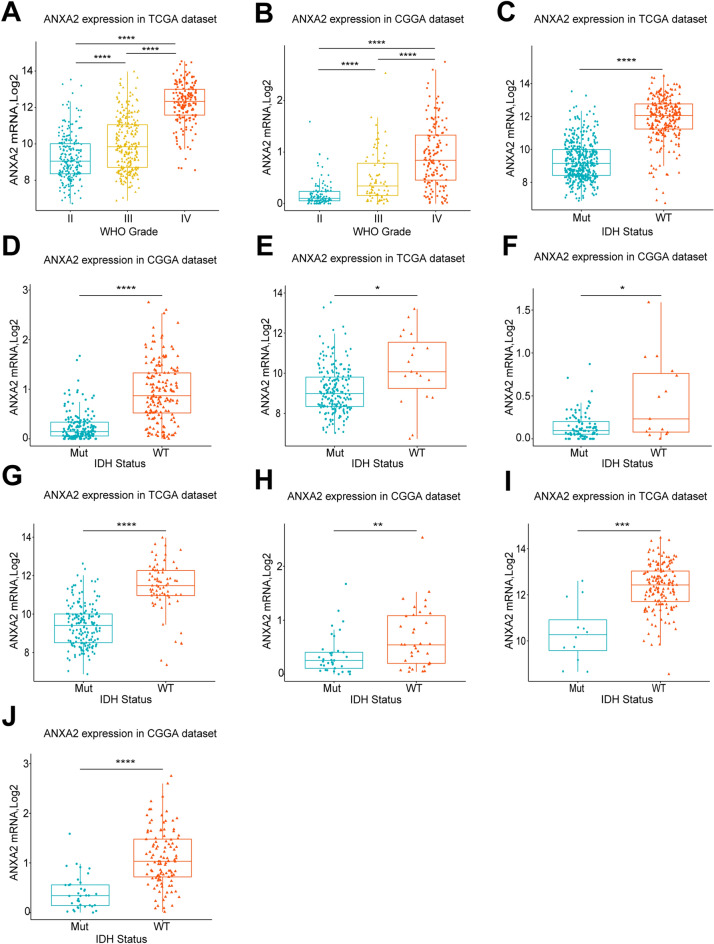
Comparison of the expression of ANXA2 in gliomas of different WHO grades and IDH statuses. ANXA2 was significantly highly expressed in HGG and IDH-wildtype gliomas in the TCGA and CGGA datasets. The expression of ANXA2 in the TCGA dataset according to WHO grade (**A**) and IDH status (overall WHO grades (**C**), WHO II (**E**), WHO III (**G**), WHO IV (**I**)). The expression of ANXA2 in the CGGA dataset according to WHO grades (**B**) and IDH status (overall WHO grades (**D**), WHO II (**F**), WHO III (**H**), WHO IV (**J**)). *P < 0.05, **P < 0.01, ***P < 0.001, **** *P* < 0.0001.

We found that the expression level of ANXA2 increased with the degree of glioma, there was a statistically difference in the expression level of ANXA2 among different grades. The expression of ANXA2 was significantly increased in HGG, especially GBM. The above conclusions were consistent in both databases (Fig. 1A,B). The identification of IDHS mutations helps to distinguish different glioma subtypes^2, 12^. Therefore, we analyzed the differences of ANXA2 in gliomas with different grades and IDH mutation types. The results showed that ANXA2 was significantly upregulated in IDH wild-type glioma in general (Fig. 1C,D), and this phenomenon was more significant as the tumor grade increased (WHO grade II, Fig. 1E,F; WHO grade III, Fig. 1G,H; WHO grade IV, Fig. 1I,J).

### ANXA2 predicts worse survival in glioma patients

**Figure 2:**
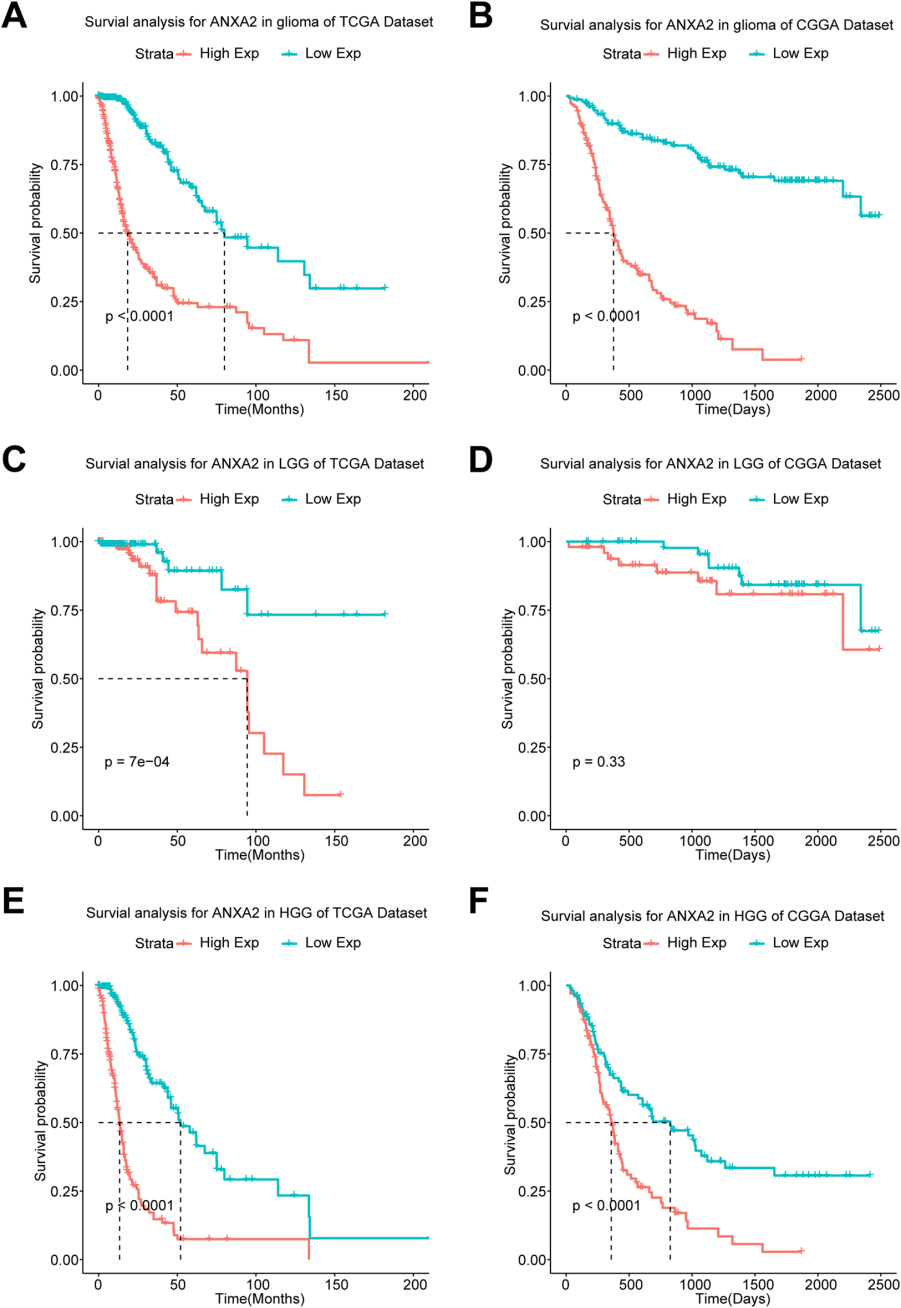
Survival analysis of glioma patients based on ANXA2 expression in TCGA and CGGA cohorts. Kaplan–Meier analysis indicated that high expression of ANXA2 was related to significantly worse prognosis overall in glioma (**A,B**), LGG (**C,D**) and HGG (**E,F**) patients.

The above results suggest that ANXA2 may be a potential indicator of glioma malignancy. Then we explored the prognostic value of ANXA2 in glioma with Kaplan–Meier curves. The results showed that the higher the expression of ANXA2 was in overall patients, the lower the OS rate was (Fig. 2A,B). To avoid differences resulting from tumor heterogeneity, we also analysed the prognostic value of ANXA2 expression in low-grade glioma (LGG) (Fig. 2C,D) and HGG (Fig. 2E,F) among patients in the two datasets, and the conclusions were similar. These results indicated that high expression of ANXA2 in glioma indicates poor patient prognosis in glioma and could be used as a negative prognostic biomarker.

**Figure 3:**
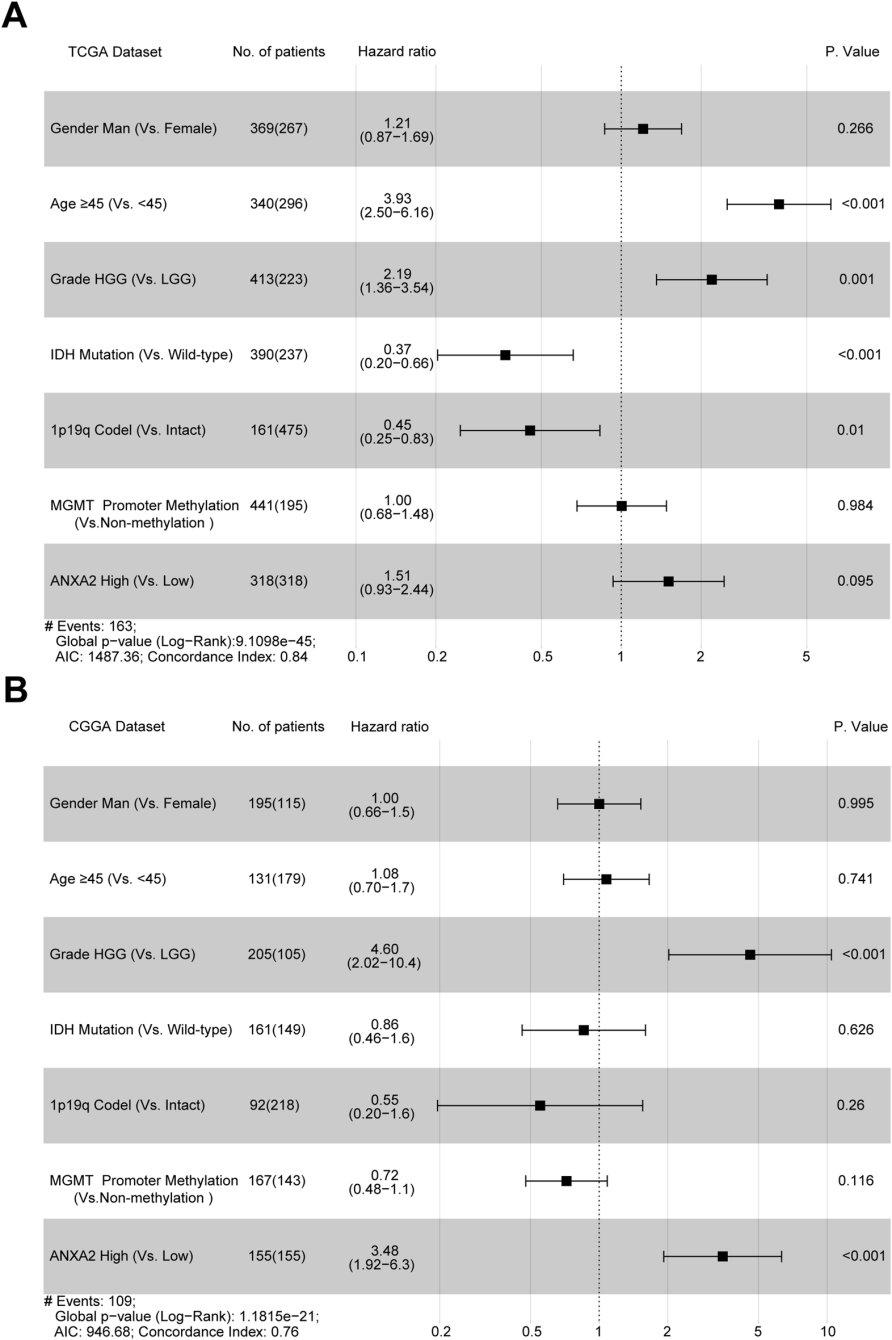
Forest plot of hazard ratios for overall survival rates assessed by ANXA2 and clinicopathological factors. ANXA2 was an independent prognostic factor after adjusting for age, grade, IDH status, 1p19q status and MGMT status in the TCGA (**A**) and CGGA (**B**) datasets.

To further investigate the independent prognostic value of ANXA2, we performed multivariate Cox regression analysis using the TCGA and CGGA datasets. After adjusting for the five clinicopathological factors (age, grade, IDH status, 1p/19q status and O^6^-methylguanine-DNA methyltransferase (MGMT) status), ANXA2 expression remained an independent prognostic factor for glioma patients (Fig. 3). Meanwhile, the minute difference in the multivariate analysis between the two cohorts may be due to the differences in the tumor grades and the intratumoral heterogeneity among these glioma samples. These findings indicated that ANXA2 conferred a poor prognosis on glioma patients, especially HGG patients.

### The expression of ANXA2 is higher in mesenchymal molecular subtype glioma

**Figure 4:**
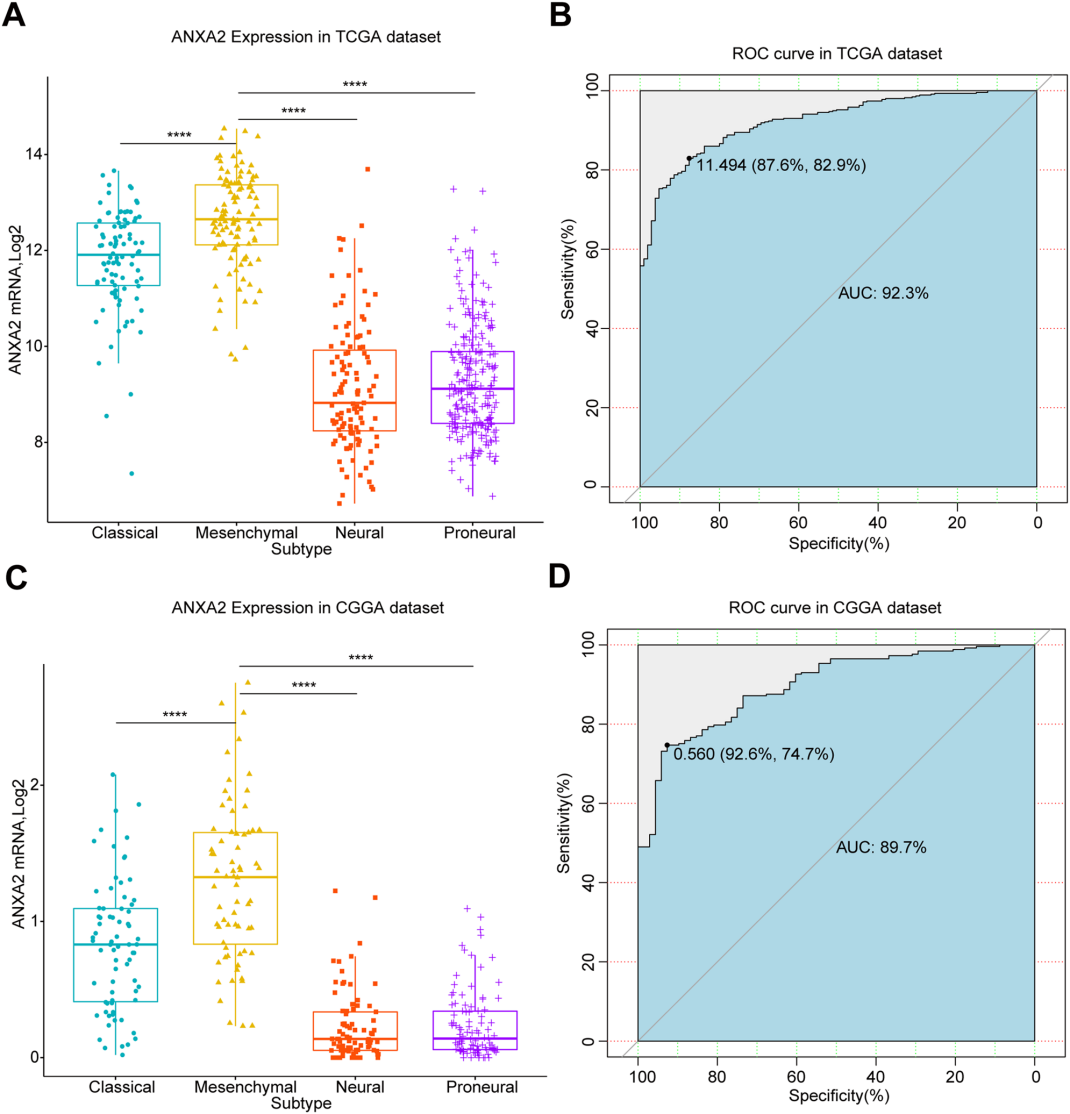
Comparison of ANXA2 expression levels in different TCGA molecular subtypes. ANXA2 was significantly enriched in the mesenchymal subtype in TCGA (**A**) and CGGA (**C**) cohorts (*P* < 0.0001). ROC curve analysis showed the predictive value of ANXA2 for mesenchymal subtype in the TCGA and CGGA cohorts (**B,D**). *P < 0.05, **P < 0.01, ***P < 0.001, **** *P* < 0.0001.

We studied the expression of ANXA2 in different TCGA molecular subtypes. In both the TCGA and CGGA datasets, the expression level of ANXA2 in the mesenchymal subtype was significantly higher than that in the other subtypes (Fig. 4A,C). Then, we conducted receiver operating characteristic (ROC) curve analysis for ANXA2 and the mesenchymal subtype for both datasets. The area under the curve (AUC) of the TCGA dataset was 0.923, and the sensitivity and specificity were 82.9% and 87.6%, respectively, at the optimal cut-off value of 11.494 (Fig. 4B). The AUC of the CGGA dataset was 0.897, and the sensitivity and specificity were 74.7% and 92.6%, respectively, at the optimal cut-off value of 0.56 (Fig. 4D). These results indicate that ANXA2 overexpression is highly specific to the mesenchymal subtype of glioma and can be used as a biomarker for predicting the mesenchymal subtype of glioma.

### ANXA2 expression is closely related to immune functions in glioma

**Figure 5:**
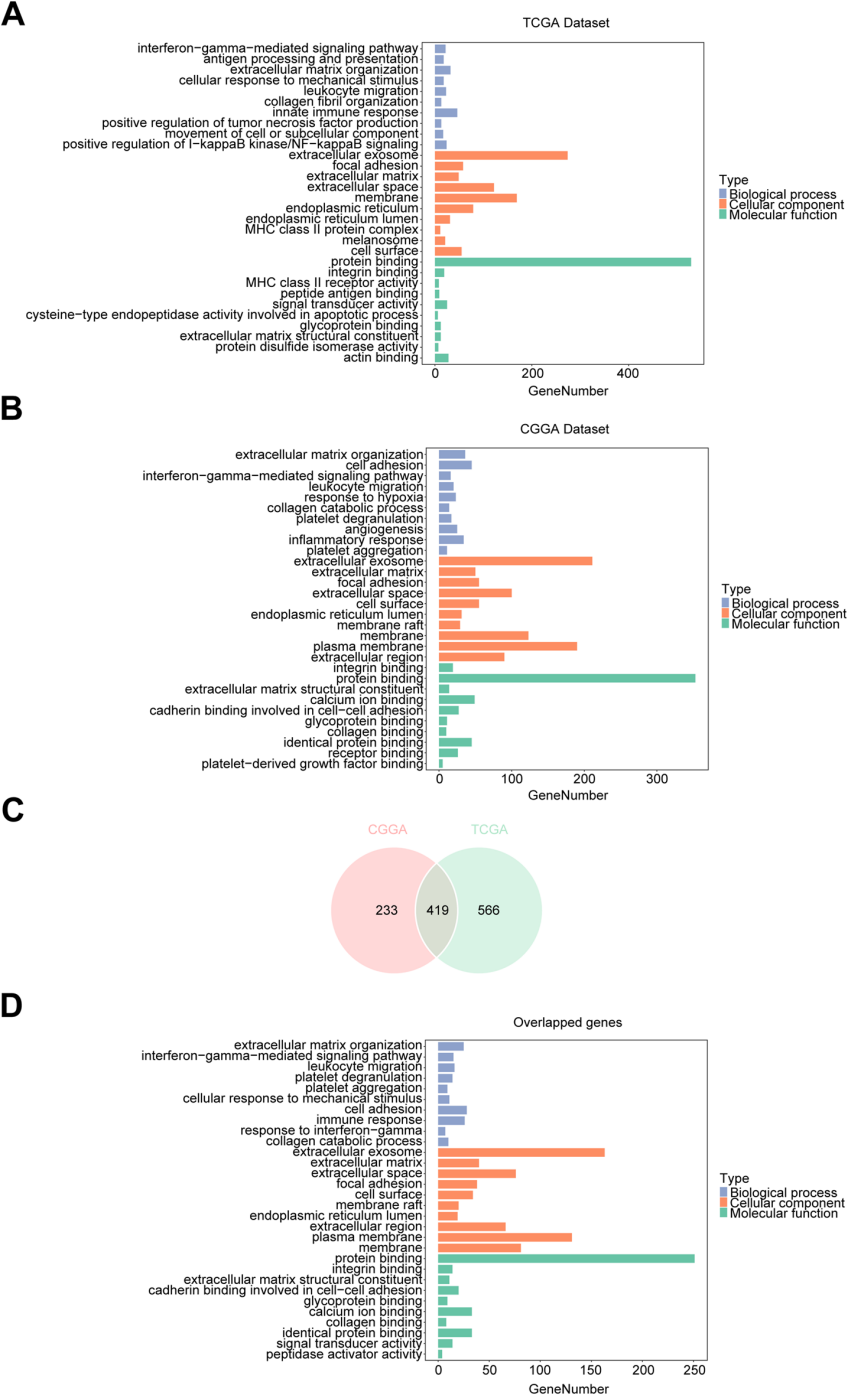
GO analysis of ANXA2-related characteristics in glioma. The results revealed that ANXA2 is involved in some important biological processes in glioma based on TCGA (**A**) and CGGA (**B**) datasets. GO analysis of 419 genes common to both datasets was used for validation (**C,D**).

To investigate the biological signatures associated with ANXA2 in gliomas, we ranked the related genes based on Spearman’s correlation analysis (|R|> 0.6 and *P* < 0.05). A total of 985 related genes (715 positively corelated genes, 270 negatively corelated genes) and 652 related genes (501 positively corelated genes, 151 negatively corelated genes) were screened out from the TCGA and CGGA datasets, respectively, as shown in Supplementary Table S1. Then, we performed GO functional analysis of these genes using the DAVID website. The results showed that the genes most associated with ANXA2 were mainly involved in the GO terms cell adhesion, innate immune response and inflammatory response, leukocyte migration and interferon-gamma-mediated signalling pathway, positive regulation of I − kappa B kinase/NF − kappa B signalling, extracellular matrix organization and collagen degradation, angiogenesis and hypoxia reaction. In terms of molecular function, the genes associated with ANXA2 significantly acted on protein binding. In terms of cell components, ANXA2 mainly affects extracellular exosomes. The above conclusions were consistent across the TCGA and CGGA datasets (Fig. 5A,B). Furthermore, we performed GO functional analysis on the 419 related genes intersecting the two datasets intersecting and found similar results (Fig. 5C,D). Our results showed that ANXA2 plays an important role in the immunobiological processes of glioma patients.

**Figure 6:**
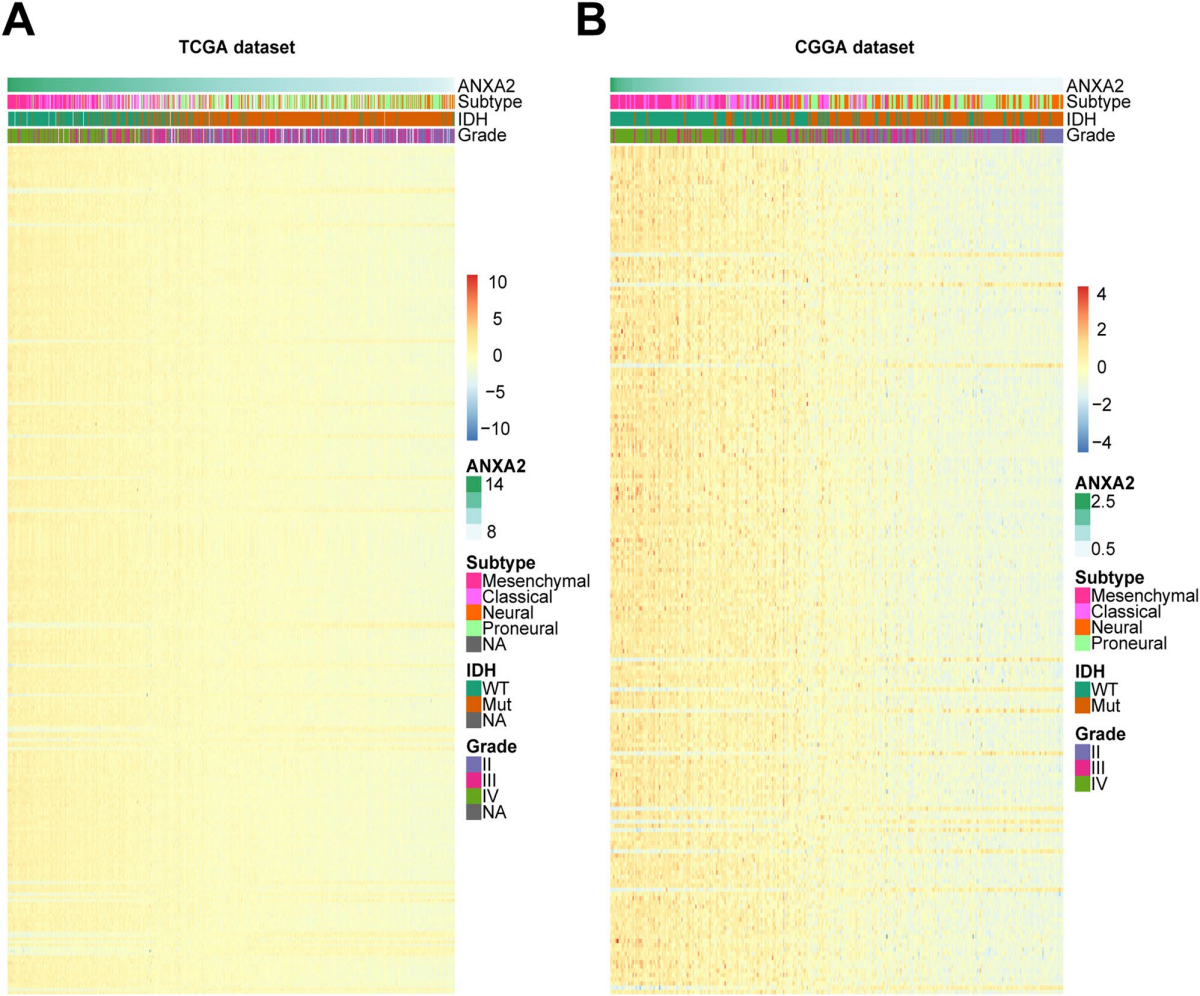
Heatmap analysis of the relationship between ANXA2 and immune function-related genes in glioma. The results showed that ANXA2 had a markedly positive correlation with most immune genes in both TCGA (**A**) and CGGA (**B**) cohorts.

To determine the immune functions of ANXA2 in glioma, we downloaded immune system gene sets from the AmiGO 2 website. Then, the immune-related genes significantly related to ANXA2 were screened from the TCGA and CGGA databases (|R|> 0.6, *P* < 0.05). A total of 285 genes in the TCGA database and 199 genes in the CGGA database were selected, and the heatmap analysis of the above immune-related genes showed that the majority of these genes were positively correlated with the expression of ANXA2 (Fig. 6). The list of these genes is illustrated in Supplementary Table S2. Our results indicate that ANXA2 expression is significantly correlated with immune function in glioma.

### ANXA2 expression is closely associated with inflammatory activity in glioma

**Figure 7:**
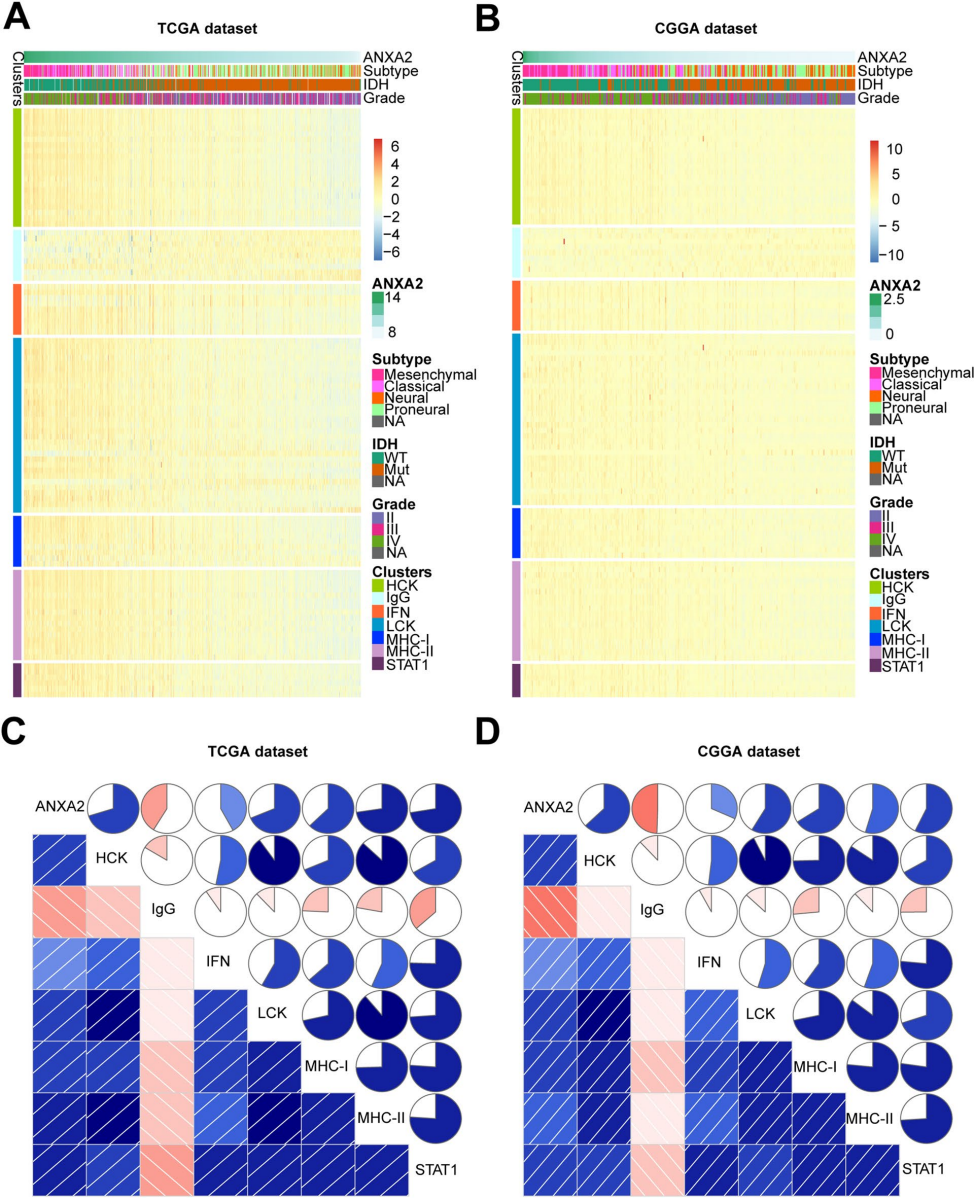
ANXA2-related inflammatory activities in glioma. The relationship between ANXA2 and WHO grade, IDH status, molecular subtypes and inflammatory metagenes are presented as a heatmap in TCGA and CGGA databases (**A,B**). Correlograms showed the correlation between ANXA2 and inflammatory metagenes (**C,D**). Blue represents positive correlations, and red represents negative correlations. Color intensity and the size of the circle in pie charts are proportional to the correlation coefficients. The results indicated that ANXA2 was significantly positively correlated with most inflammatory activities.

According to the results above, ANXA2 is mainly involved in the inflammatory response in glioma. To further investigate the role of ANXA2 in inflammatory activity in glioma, we included 104 inflammatory and immune response-related genes to generate seven metagenes^13^. Supplementary Table S3 summarizes the detailed list of these genes. Based on the TCGA and CGGA datasets, we performed heatmap clustering analysis on the above genes and found that all of the metagenes except IgG were positively associated with ANXA2 expression (Fig. 7A,B). To validate the clustering results, we applied correlograms according to the Pearson correlation between ANXA2 and the seven metagenes (Fig. 7C,D), and the result was highly consistent with the above heatmap analysis. Therefore, we found that ANXA2 was significantly positively correlated with HCK, MHC-II, STAT1, LCK, MHC-I and interferon in the TCGA and CGGA cohorts but negatively associated with IgG.

### Relationship between ANXA2 expression and tumor-infiltrating immune cells

**Figure 8:**
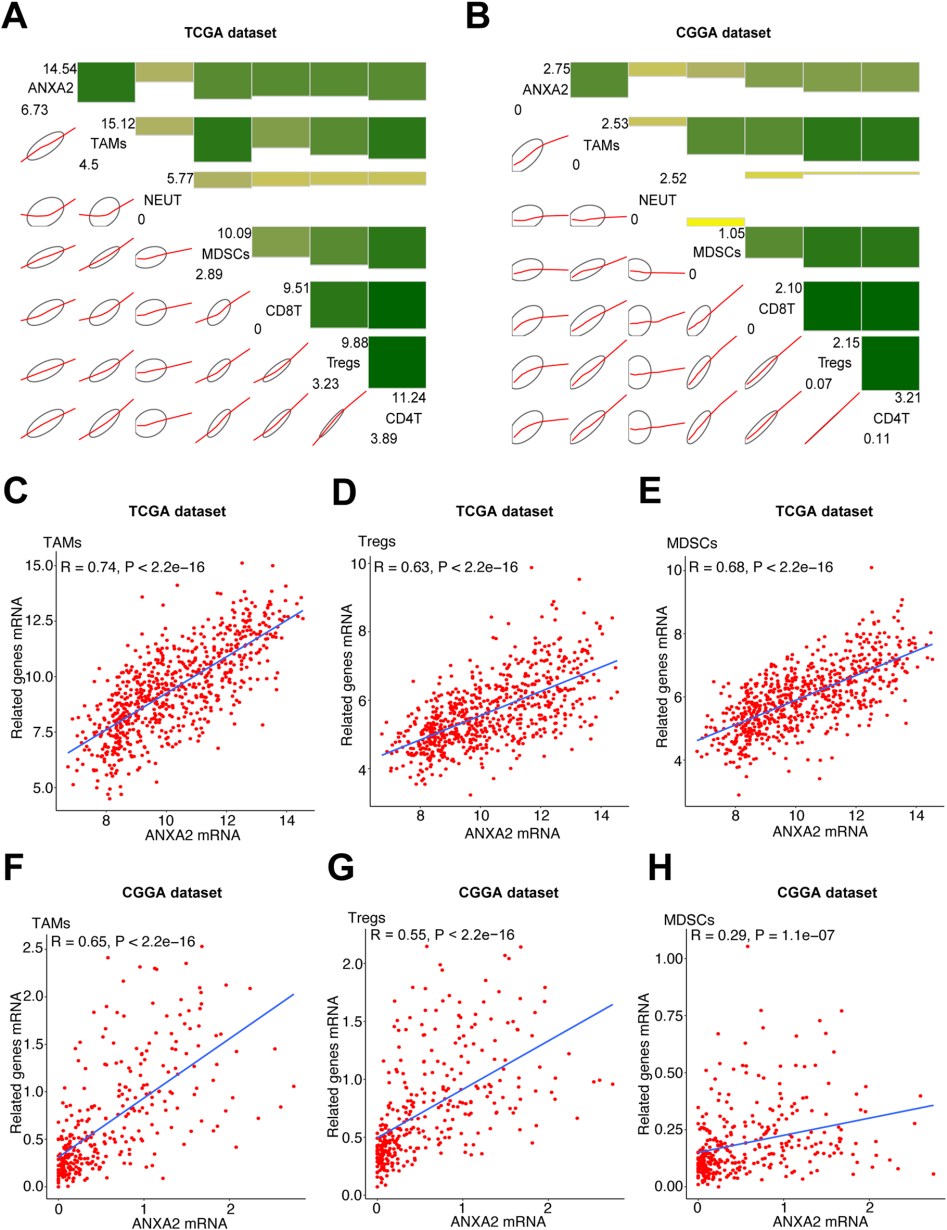
ANXA2-related infiltrating immune cells in glioma. (**A,B**) Correlograms showing the correlation between ANXA2 and immune cell infiltration level based on both datasets. Green represents positive correlations, and yellow represents negative correlations. Color intensity and the size of the bars and ellipses are proportional to the correlation coefficients. The leading diagonal contains the minimum and maximum values of variables. (**C–H**) ANXA2 was significantly positively correlated with TAMS, Tregs and MDSCs in both datasets. Each point represents a glioma sample. A regression line was fitted to the dot plot.

Tumor-infiltrating immune cells play a key role in tumor development and control. Thus, we examined the correlation between ANXA2 and immune cell-specific marker genes. Six immune cell types that frequently infiltrate tumors were selected, including CD4 + T cells, CD8 + T cells, regulatory T cells (Tregs), tumor-associated macrophages (TAMs), myeloid-derived suppressor cells (MDSCs) and neutrophils. Detailed information about the immune cell-specific marker genes is listed in Supplementary Table S4. As shown by correlation analysis, glioma-derived ANXA2 expression was positively correlated with biomarker gene expression in all six immune cell types in the TCGA and CGGA datasets (Fig. 8A,B). These immune cells may be important effector cells in inflammation and the immune response. These results are also consistent with the results of GO analysis. We also found that the expression of ANXA2 was positively correlated with TAMs, Tregs and MDSCs in both the TCGA and CGGA datasets: TAMs (r = 0.74 in the TCGA dataset, r = 0.65 in the CGGA dataset; Fig. 8C,F), Tregs (r = 0.63 in the TCGA dataset, r = 0.55 in the CGGA dataset; Fig. 8D,G), and MDSCs (r = 0.68 in the TCGA dataset, r = 0.29 in the CGGA dataset; Fig. 8E,H). Therefore, our results suggest that glioma with high expression of ANXA2 tends to have more infiltrating immune cells, especially immunosuppressive immune cells.

## Discussion

This study is the largest large-scale clinical study of ANXA2 in glioma thus far, and we comprehensively analyzed the expression pattern and related immune characteristics of ANXA2 and its clinical significance. First, we proved that the expression of ANXA2 was significantly upregulated with increasing WHO grade, and similar expression patterns were also reported in previous studies^5, 11, 14^. We also found that ANXA2 is highly expressed in known malignant glioma molecular phenotypes, such as IDH wild-type and mesenchymal subtype gliomas. Mesenchymal GBM cells were regarded as the most aggressive^15^, and we found that ANXA2 is a potential predictor of mesenchymal subtype. These results indicated that ANXA2 expression was closely corelated with the malignant biological process of glioma, and patients with higher levels of ANXA2 expression have a greater risk of tumor recurrence, progression, and therapeutic resistance than other patients. The results of the survival analysis showed that the overall survival time of whole-grade glioma and HGG patients with high expression of ANXA2 was significantly reduced, which was consistent with the findings of previous studies^11, 16^. In addition, multivariate Cox regression analysis suggested that ANXA2 was an independent risk factor for the clinical prognosis of glioma patients, which further indicated that ANXA2 was a negative marker for the prognosis of glioma and could be used for the molecular biological diagnosis and evaluation of glioma. The value of ANXA2 as a biomarker to diagnose tumors has previously been reported in liver cancer and lung cancer; for example, ANXA2 has been used as an immunosensor for lung cancer^17^ and has been used for the early diagnosis of liver cancer via enzyme-linked immunosorbent assay (ELISA)^18^. ANXA2 can also be used to monitor EMT in ovarian and breast cancer^19^. We confirmed that ANXA2 can be used as a molecular marker for the clinical diagnosis of glioma and can be used to evaluate the prognosis and outcomes of glioma patients through large-scale clinical studies. ANXA2-based treatment may be an important potential strategy for the comprehensive management of glioma.

In this study, we also analyzed the biological function of ANXA2 in glioma. GO analysis showed that ANXA2 was involved in the glioma immune response, inflammatory response, neovascularization, fibrin decomposition and other processes. The increased expression of ANXA2 and S100A10 leads to increased plasmin production, which leads to the degradation of the extracellular matrix and activation of matrix metalloprotein, allowing tumors to invade surrounding organs or local blood vessels^20^. ANXA2-mediated proteolytic enzyme activation and tyrosine phosphorylation are key drivers of new angiogenesis, proliferation, invasion and metastasis^5^. ANXA2 promotes neovascularization as a regulator of fibrinolytic protein production on the cell surface^21^. The ANXA2 heterotetramer is an assembly site for plasminogen and tissue plasminogen activators on the surface of endothelial cells, thereby promoting plasminogen production and clearing fibrin formed on the surface of blood vessels, facilitating the response to more subtle vascular damage^22^. These results suggest that ANXA2 may promote tumor progression and invasion by increasing the tumor blood supply and promoting the degradation of extracellular matrix around tumors. Therefore, the combination of ANXA2-targeting and antiangiogenic therapy may be an effective treatment for glioma.

High expression of ANXA2 in the tumor microenvironment has been shown to be involved in local immunosuppression and immune escape^5^. Current studies suggest that glioma also has significant immunosuppression and immune escape, which plays an important role in its progression and resistance to treatment^3^. For example, gliomas can activate microglia to suppress immune responses (phagocytosis)^23^. However, the role of ANXA2 in glioma immune escape remains unknown. Therefore, we conducted enrichment analysis and correlation analysis of ANXA2-related genes and found that ANXA2 was closely related to tumor-related immune responses and inflammatory activities, and these results were consistent with the results of the GO analysis. Additionally, it was found that immune cell infiltration was significantly correlated with the expression of ANXA2, especially the infiltration of TAMs, Tregs, and MDSCs, suggesting that ANXA2 may be an important factor involved in glioma immunosuppression and tumor progression and invasion in the inflammatory microenvironment of glioma. This is a new discovery. All these results suggest that it is possible to use ANXA2 as a target for the treatment of glioma, which may reduce the immunosuppression in glioma and improve the overall prognosis of glioma patients, especially HGG patients. Previous studies have shown that ANXA2 is also involved in immunosuppression in renal cell carcinoma^24^. ANXA2 can promote an increase in proportion of Treg cells and the expression of some immune checkpoint molecules, while reducing the proportion of natural killer cells and dendritic cells (DCs) and the expression of some inhibitory molecules, contributing to the immune escape of tumors^25^. The interaction between ANXA2 and DC-specific intracellular adhesion molecule (ICAM)-3 grabbing nonintegrin (DC-SIGN, CD209) resulted in immunosuppression in nasopharyngeal cancer^10^. A recent study has shown that the actin cytoskeleton regulated by ANXA2 has a negative effect on T cell aggregation, which may be one of the mechanisms by which upregulation of ANXA2 expression in tumors leads to reduced T cell activation and immune imbalance in the tumor microenvironment^26^. Further study of the role of ANXA2 in immunosuppression will be helpful for the development of ANXA2-targeted immunotherapy^5^.

Some researchers have suggested that ANXA2-targeted therapy can help improve the poor therapeutic response associated with high ANXA2 expression in cancer^10^. In recent years, targeted treatment of ANXA2 has been reported via in vivo experiments in animal models for a variety of tumors, such as breast cancer ^27^, ovarian cancer^28^, pancreatic cancer^29^, teratoma^30^ and others. Furthermore, ANXA2 can activate the Vγ8Vδ3 T cell receptors specifically and is identified and targeted as an antigen by the FMS-01 monoclonal antibody, which specifically inhibits Vγ8Vδ3 TCR-mediated recognition of GBM cells^31^. This antibody can also be used as a vaccine adjuvant to enhance the effectiveness of glioma vaccines^32^. Furthermore, ANXA2 antibody can inhibit the migration, invasion and proliferation of primary GBM cells^33^. Previous study proved that ANXA2 knockdown in rodent glioma GL261 cells reduces migration in vitro, slows tumor growth, invasion, proliferation, angiogenesis and increases tumor cell death in vivo^34^. Recent studies also showed that ANXA2 knockdown inhibits proliferation and invasion of canine GBM cell lines^35^, human glioma U251, U87^36^ and U118 cells^37^, primary patient-derived glioma cells^33, 35^ and GBM stem-like cells^33, 35^. ANXA2 overexpression obviously promotes invasion and proliferation of U118 cells^37^ and primary GBM cells^35^. These studies provide theoretical support for ANXA2-targeted therapy of gliomas at the cellular level, thus strengthening our conclusions from TCGA and CGGA datasets which are multi-cellular results at the tissue level rather than cell specific. In addition, further studies about the exact effects of ANXA2 on prognosis and immunosuppression for gliomas via single-cell RNA sequencing are currently in progress. The clinical application of ANXA2-targeted therapy, especially for gliomas, also needs to be further studied in more depth in the future.

In summary, this study comprehensively explored the expression pattern and biological function of ANXA2 in glioma through large samples. We found that high expression of ANXA2 predicted malignant pathological subtypes of glioma and poor patient prognosis, and that ANXA2 expression was closely related to the glioma-related immune response, especially inflammatory activity and immunosuppression. Combination and individualized therapy will play an important role in the future^3^, and ANXA2-targeted immunotherapy alone or in combination with other therapies is expected to become a new future therapeutic strategy for glioma patients.

## Method

### Patients and samples

All the RNA-seq data and clinical data of glioma patients analyzed in this study ranging from WHO II to IV were collected from two independent databases, the TCGA database (699 glioma samples) (http://cancergenome.nih.gov/) and the CGGA database (325 glioma samples) (http://www.cgga.org.cn). The glioma samples from the CGGA database were used to avoid the limitations of a single-database study. Gene expression profiling data were log-transformed for further analysis. We excluded 15 samples and 63 samples from the TCGA and CGGA databases, respectively, which did not have available RNA-seq data, molecular pathological information or useful overall survival (OS) rate information. This research was approved by the Ethics Committee of the Peking University Third Hospital.

### Statistical analysis

All the statistical analyses and figures were generated with R software for MacOS, version 4.0.3 (http://www.r-project.org). The overall survival rate difference was calculated with the Kaplan–Meier method. Cox regression analysis was performed with the survival package in R, and other R packages, such as ggpubr, ggplot2, devtools, pheatmap, pROC, corrplot and corrgram, were used to generate figures. The genes significantly related to ANXA2 were filtered out via Spearman’s correlation analysis. Gene ontology (GO) functional analysis to identify enriched biological processes and functions was performed using DAVID Bioinformatics Resources 6.8 (https://david.ncifcrf.gov/). Gene sets of the immune system were downloaded from the AmiGO 2 website (http://amigo.geneontology.org/amigo). Pearson’s correlation was used to determine significant differences. One-way ANOVA was used to test for differences among at least 3 groups. Student’s t-test was used to determine differences in each 2-group comparison. All differences were considered statistically significant at the level of P < 0.05.

## Ethics approval

This research was approved by the Ethics Committee of the Peking University Third Hospital (S2020018).

## Supplementary Information

Supplementary Information 1.

Supplementary Information 2.

Supplementary Information 3.

Supplementary Information 4.

## Data Availability

The datasets presented in this study can be found in online repositories. The names of the repository/repositories and accession number(s) can be found in the article/ Supplementary Material.

## Abbreviations

CNS: Central nervous system
GBM: Glioblastoma
EMT: Epithelial mesenchymal transformation
HGG: High-grade glioma
LGG: Low-grade glioma
OS: Overall survival
TCGA: The Cancer Genome Atlas
CGGA: Chinese Glioma Genome Atlas
ROC: Receiver operating characteristic
MGMT: O^6^-methylguanine-DNA methyltransferase
AUC: Area under the curve
IDH: Isocitrate dehydrogenase
GO: Gene ontology
TAMs: Tumor-associated macrophages
MDSCs: Myeloid-derived suppressor cells
DCs: Dendritic cells
Tregs: Regulatory T cells
ELISA: Enzyme-linked immunosorbent assay

## Code availability

Custom code used to analyse the RNA-seq data and datasets generated and/or analysed in the current study are available from the corresponding authors upon request.
