# Supplementary material for: ANXA2 is correlated with the molecular features and clinical prognosis of glioma, and acts as a potential marker of immunosuppression
Source: Sci Rep. 2021 Oct 21;11:20839. doi: 10.1038/s41598-021-00366-8 (PMC8531374; doi:10.1038/s41598-021-00366-8)
Supplement: Supplementary file 1 — Supplementary Information 1. [file 41598_2021_366_MOESM1_ESM.pdf]

ANXA2 is correlated with the molecular features and clinical prognosis of glioma, and acts as a potential marker of immunosuppression

Kaiming Ma[1,2], Xin Chen[1,2], Weihai Liu[1,2], Yang Yang[1,2], Suhua Chen[1,2], Jianjun Sun[1,2], Changcheng Ma[1,2], Tao Wang[1,2], Jun Yang[1,2*]

[1] Department of Neurosurgery, Peking University Third Hospital, Beijing, China.

[2] Center for Precision Neurosurgery and Oncology of Peking University Health Science Center, Beijing, China.

*** Correspondence:** Jun Yang

Address: Department of Neurosurgery, Peking University Third Hospital, 49 North Garden Rd, Haidian District, 100191, Beijing, China.

Email： bysysjwk@126.com

Table S1. Genes associated with ANXA2 in TCGA and CGGA datasets.

Genes in TCGA dataset	Genes in CGGA dataset
GABBR1	ZDHHC22
ZFYVE20	RASL10A
PIP4K2B	SHISA7
KIAA0427	CASKIN1
ZDHHC22	ENHO
JPH4	RUNDC3A

JPH3	LRRC4
MTA3	BAI3
KIF3A	DUSP26
HDAC4	NTNG2
BAI3	FAM57B
DNM3	LOC100506548
MAPT	SLIT1
CDHR1	GNAO1
SPTBN2	DSCAML1
TMEM170B	GLUD1
NAP1L3	FLRT1
SHANK2	LOC100132273
TUB	STX1B
ZC3H12B	TMEM151B
UNC80	RALGPS1
TTC3	SERP2
ATP8A1	PRDM11
GABRB3	ABCC8
CPEB3	GABBR1
CASKIN1	FRY
SATB1	HMP19
MADD	ALDOC
RUNDC3A	SCG3
CRY2	MCF2L2
SRGAP3	TEF
FAM155A	KCNB1
BZRAP1	OMG
UNC13A	NAP1L3
NDRG3	CRHR1-IT1
REPS2	LINC00634
BCL7A	PHYHIPL
PDZD4	ADHFE1
CRTC1	CDK5R1
TOM1L2	LOC100287846
NRXN1	GPM6A
ZNF25	ZC3H12B
CDK5R1	PDZD4
KIF21B	LOC254559
C12orf51	GPR123
MAP2	SMPD3

GRIA2
TEF
GNAO1
LRRC4
MLLT6
KIAA1409
FAM190B
SHISA7
KCNIP3
TNRC6C
POU6F1
SGSM1
CEP68
FAM168B
SMPD3
PLCB1
GDAP1
MGEA5
SLITRK5
DOK6
KIAA0430
DLGAP1
CLASP2
RALGAPA1
PAFAH1B1
ELFN2
TNK2
PRKCE
ARHGEF9
NALCN
PDE2A
ALDH5A1
BTRC
FASN
ZRANB1
SLC25A27
FLRT1
KCNJ9
GNAL
CRTAC1

TNK2
UNC79
NRXN2
NALCN
PTCHD2
UNC5A
FAIM2
STARD10
CDH20
CEP68
DOK6
NOG
DNAJC18
CXXC4
FLJ22184
MCMDC2
MLLT6
MAST1
BSN
GRID1
CNRIP1
THRA
PHLPP1
SPTBN2
TNR
ZFYVE20
C22orf39
TSPAN7
C17orf96
LINGO1
HLF
TPTE2P1
SSTR2
INA
SLITRK5
LMF1
B3GALT2
PRKCE
ARL3
ATP9A

SCAMP5	CPLX1
HERC1	GDPD1
DUSP26	HDAC4
42250	FAXC
CHGB	SOX8
MTMR9	CSMD1
MAST1	CRY2
LPHN1	HECTD4
TMEM151B	ATCAY
C17orf96	LINC00641
SREBF2	RAB6B
STX1B	LRRC37B
UNC5A	NRSN1
TRIM23	CSDC2
ATP6V1G2	TMEM170B
MYST4	TSPYL4
PLEKHM3	MTMR7
CECR6	FUT9
MCF2L2	ADARB2
KCNIP2	ACVR2B
CAMSAP1L1	FCHSD2
JMY	SLC22A17
CUX2	DOCK3
TERF2	LINC00672
FRY	PTPN4
USP27X	KCNJ11
USP11	ARNT2
ZNF248	UNC13A
DGCR9	SNX32
ANKRD26	SLC6A1
ZNF238	MTSS1L
MAPK8IP2	FAM171A1
ACVR2B	CHRNB2
TMOD2	ANKRD16
INA	FP588
SCAI	CHGB
TSPYL4	GALNT13
RALGPS1	RFTN2
BRSK2	SYNGR1
SLC6A1	DGCR9

BSN	KIAA1107
GPR123	HIP1R
RUSC2	SYP
NTNG2	TMEM35
PHYHIPL	RTN4R
HMP19	C14orf132
ZNF33A	AKAP6
HNRNPUL2	OPHN1
PLA2G6	MAP2
ATP9A	DGCR6
FCHSD2	PIP4K2B
PPP1R9A	FAM222A
FAM57B	TBKBP1
WASF1	LRRC16B
ACACA	MN1
KIF3C	NECAB2
RAP2A	FBXL16
CNRIP1	FAM19A2
ZC3H7B	FLJ42627
FAM13C	KIF3A
LRRC20	ZNF25
FAIM2	KCNJ9
CACNA2D2	LSAMP
GDPD1	ABAT
SBF1	DLGAP3
SCN3B	HEBP2
PANK1	CTTN
DOCK3	TMEM37
C6orf168	PTGER4
PSD	PAM
NRXN2	RHOQ
SCG3	IL1R1
NCAM1	TMEM43
FAM117B	PSME2
GRIA4	TGFB1
PIK3R1	PI4K2B
C6orf174	CD74
GRID1	NDUFA4L2
FRMPD1	HIST1H2BK
AKAP11	LXN

DGCR2
PHLPP2
AKAP6
NMNAT2
RAB11FIP2
LHFPL4
LRRC37B
CHRNB2
RICTOR
DSTYK
LRRTM4
SCAPER
TSC1
WDR37
UBR3
MTMR7
OMG
ATCAY
LRRC16B
CYFIP2
ZBTB44
R3HDM2
DZIP3
RAPGEF4
CNTNAP2
NOG
ARHGAP32
ATP6V0A1
UBE2O
PLCXD2
SSTR2
LSAMP
MLL
RTN3
HSFX2
GRIN3A
FAM126B
TMEM63C
MAPK8IP3
SMARCC2
CREB3L2
TRIP10
NAGA
RFTN1
CCL2
TNFRSF10D
MFSD7
SEC22B
TMEM154
ARL4C
ZC3H12A
CTSB
LIMK2
SEMA3F
CNPY4
PSMC2
SPRY1
MACC1
HLA-DQB1
LACTB
PSMB9
FHL3
CD300A
CYSTM1
EPHA2
CD276
ZMPSTE24
TNFAIP2
EIF4G2
PHTF1
CDR2
TNFRSF10C
IFITM2
HSD3B7
WDR1
XKR8
ICAM3
RAB13
FXYD5
EIF4E2

SYNGR1
PVRL1
ASB1
OPHN1
FAM110B
GFRA1
TARSL2
RUFY2
FAM171A2
RNF165
KBTBD6
DOPEY1
SLC8A2
SEMA6B
DEPDC5
HLF
UBQLN2
TMEM136
RAPGEF2
ACADSB
LARGE
NRSN1
ZCCHC14
RIC3
NAP1L2
NEURL4
ZMYND11
MAGEE1
SYT15
ZNF711
MYCBP2
AKT3
FAM192A
LOC283267
RUFY3
SOX8
DENND5B
LOC339674
PID1
TTBK1
AHR
CELSR1
SNTB2
MTMR11
COL6A1
FAP
ACAP1
ACTB
TLR2
ABRACL
ADAM19
ADAMTS1
EFNB2
IL7R
IL13RA1
DIRAS3
CABP4
C12orf5
ACTA2
FAM126A
FKBP10
CAV1
MUL1
SMS
STAB1
HDAC1
RILPL2
SH2B3
APLN
ELK3
SECTM1
GMPPA
SDF4
TPM4
CPQ
PTGFRN
HLA-DMA
LAIR1
EDEM1
ENPEP

KIF1A	ITGA1
EVI5L	CEBPD
NF1	HMGCL
RAB11FIP4	ACTN4
TTLL12	TXLNA
EIF4ENIF1	B3GNT5
CHD3	HLA-DPB1
SIK3	ARHGAP15
FXR2	LRRN4CL
RB1CC1	C5orf15
SLIT1	TRIM21
SEZ6L	PELO
CTTNBP2	TRPV2
GIT1	FOSL2
GRIPAP1	ZDHHC5
GRAMD1B	FRMD8
AMZ1	SHQ1
SPIN3	FZD1
ANKRD46	PION
SS18L1	CTSD
EML5	C1QTNF6
GPR158	BAK1
KIAA1147	SAMD9L
ABAT	ARF4
SLC25A19	YKT6
ARAP3	TNFRSF1B
CCRL2	FAM115C
CTF1	HCLS1
LMO2	FTSJ2
NME2	FBLN5
MGAT4B	ARID5A
FAM111A	GBE1
PMM2	TEAD3
SLC25A39	MYL9
NUDT16P1	LBX2-AS1
SLC26A2	FUCA1
ST14	ANPEP
ADPRHL2	DNAJC3
SOCS2	ABHD15
PSMA7	TMCO4

SLFN12
CLEC18A
PYCARD
TM4SF1
C8orf76
DNALI1
ZFP36L2
TCEA3
GGCX
PSRC1
CFH
BTN2A2
XKR8
RPS2P32
WEE1
BATF3
SPRY1
RNPEP
ATF3
HM13
TRNAU1AP
GBGT1
SERF2
GNAI3
IL13RA1
B3GNT7
PTGS1
CTSL1
PROS1
TRAPPC3
MANF
MLKL
RPA3
LHFPL2
CHPF2
ROBLD3
HIST1H2BK
SEC61B
ACTR3
TAF12
PRDX4
AAED1
GNG5
ICMT
HLX
ZDHHC18
DDB2
ST14
ZBTB8OS
NFAM1
FADD
KDELC2
TWF2
COTL1
THBS1
LMAN2
CASP6
NEAT1
CHST2
PRKCDBP
BST2
MB21D1
CTNNA1
CTBS
PSMB8
ZNF600
MANF
PSMD9
TRAM2
ORMDL2
FERMT3
CIDEB
RGS16
TWSG1
WWTR1
MGP
EFEMP2
GCLM
ARPC5
ADM

TIFA	MARVELD1
TTC38	DPP4
A2LD1	TMBIM1
SEC61A1	NUDT19
ATP6V0E1	HEXA
BRI3	PLAT
TWSG1	DNAJB1
CTHRC1	PMM2
SNAI1	MAP3K6
CMTM7	HLA-DRA
SLC35D2	CDCP1
PDIA6	FCER1G
TGFB1	KCNE4
SOD3	SH3BGRL3
LOC440957	ERI1
IQGAP2	GLIPR1
HSPG2	ATP8B1
PPP1CA	PPIB
DNAJB1	COPZ2
C14orf142	CRIP1
PION	IGFBP4
TMEM8A	TMEM8A
PPIC	LHFPL2
SLC2A9	EHBP1L1
SOCS1	CTSL1
PSMC4	SOD2
BOLA3	SLC12A7
C7orf42	CFB
CROT	CD58
BAK1	SFRP4
GPNMB	COL15A1
ARL11	RPN1
LAPTM5	STAT5A
RGS16	FABP5
OSCAR	MCAM
SASH3	ATF5
CD68	SLC2A10
HBXIP	SPATS2L
BGN	DNAJB11
RHOQ	LCP2

POLR2L	SLC17A9
HLA-C	SLC25A43
MRPS12	BCL10
IFI44	DPH3
PRR24	GNG12
APOL4	CXCR4
ESYT1	ETHE1
SUMF2	SHKBP1
SSR3	FN1
GEMIN7	OLFML2B
COTL1	DAP
C1QC	MAN1C1
FKBP9	ZAK
HCP5	ADAM9
TMEM49	SCPEP1
ALOX5	PPCS
LCP2	ZBTB42
DTX3L	SLAMF8
HEATR2	TRADD
ECE1	ARF6
SWAP70	MYO1F
CXCR4	GADD45A
SLC16A4	RNF149
NOD1	RAP2B
EFNA4	IL2RG
MXRA8	CLIC4
KTI12	DERL2
IL4I1	GALM
WDR78	HSPG2
EIF3I	PLOD2
TRIM21	LOC100505812
RFC2	RBMS1
CD97	B2M
LYN	FTL
MUC1	DOK3
ATG4A	SEC61A1
S100A9	C5AR1
NECAP2	HSPB1
MMP11	CYBA
TMEM107	PPP1R15A

CD14
NAT1
CPVL
PPP4C
RUNX3
SLC2A10
TXNDC5
TMCO4
XBP1
LAMP3
SUMF1
TMEM176B
TPM3
BAX
TMEM217
C17orf87
C10orf10
ABHD15
RPS6KA1
HSPA5
FERMT3
POP4
LOXL3
CRYZ
RER1
MYL12B
ITPRIPL2
C14orf119
MUL1
TMEM37
CCDC46
MYO1C
P2RY6
C19orf40
FLNC
SHKBP1
ARPC2
GMPPA
PLOD3
HDAC3

TWISTNB
ETV6
LOXL1
PLOD3
MIR22HG
FCGRT
LGALS3BP
RARS
ZDHHC12
B4GALT1
ITGA4
FCGBP
ICAM1
CSDA
GBP2
S1PR3
DTX3L
ST8SIA4
PECAM1
PDCD1LG2
FLNA
DDOST
MBD2
ARSJ
FAM46A
NPC2
BGN
LYZ
PDIA5
CFH
VMP1
PROS1
PYGL
APOBEC3F
ARHGDIB
KDELR1
SLFN12
FHOD1
NUCB1
SIGLEC9

TEAD3	KCNE3
TAX1BP3	SERTAD1
COL5A1	KCTD9
C10orf11	THBD
TM9SF1	SLC11A1
MLX	NTAN1
CD300LF	REEP4
C7orf68	TMSB10
DOK1	SERPINA3
FBLN5	DPY19L1
CP	F11R
TMEM60	HMOX1
TGFB2	GUSB
ZDHHC18	FUCA2
CSDAP1	TNC
BST2	CHSY1
TXLNA	IFITM3
ST8SIA4	TMOD3
GALNT2	LAP3
CD300A	LINC00346
HDAC1	TRIM38
C13orf18	TMEM255B
RARS	C1S
LOC154761	CTSC
CIITA	TRAM1
IGF2BP2	TOR4A
COPZ2	LOXL3
CRIP1	LTBP2
AEBP1	LRRC25
TGFBI	FAM109B
DTX2	VASN
HEXA	CD248
TSPO	NUAK2
GLRX	CMTM6
PARP12	C19orf10
HLA-DQA1	MXRA5
CLEC7A	CLEC2B
LAMB2	SAT1
LAIR1	BCL3
C1QA	ZNF217

PROCR	HTRA3
LMNA	PFN1
PGCP	TMEM109
PSMD9	APOBR
SEPN1	VAMP8
CD93	SSR3
TMEM50A	EMILIN2
GIMAP2	APOBEC3G
PSMA5	S100A10
APOL1	PVRL2
PPP1R3B	RCAN1
PPIL5	CYTIP
CTSS	SWAP70
CYP2S1	IRF1
CALD1	ADPRH
C5AR1	EVA1B
CDK2	PTX3
PLEKHA9	HLA-A
CYTIP	HSP90B1
IRAK4	MICALL2
PTGER4	GMFG
C9orf89	NOD1
EHD4	TAGLN2
GAL3ST4	TCIRG1
LRRC42	TRIP6
NUDT19	C1orf54
LUM	TNFRSF14
ERP44	VAMP5
GNS	ZMYM6NB
C1QB	SLC26A2
UBE2A	KDELR2
LOXL1	AIM1
PSMB9	MYL6
CAV1	GALNT2
TBC1D10C	SUSD2
SPP1	VKORC1
SPINT1	PCOLCE
COL3A1	SMIM3
CASP7	GBP1
RHOH	RCN3

TBC1D1
TRADD
CEBPD
BTG3
TYROBP
EHD2
SEC24D
CFB
MGP
C1orf91
CCL2
PTPN2
ARID5A
C2orf29
TMEM179B
HPS3
HLA-DOA
TEAD2
KDELC2
PPIB
SFRP4
TXNDC17
RAP2B
YIPF1
FPGT
PSMB8
CSTB
SEC61G
FMOD
SUSD2
LDHA
CAP1
C19orf10
WDR77
MARVELD1
C1orf144
RAB8A
CALHM2
PALLD
LGALS3BP
LAMB2
PROCR
ADAMTSL4
EHD4
MMP11
RRAS
ACTN1
RAC2
IKBIP
APOBEC3C
HLA-B
PLOD1
GPR65
CD151
CD93
IL10RB
SYDE1
HSPA6
GLT25D1
CALR
EHD2
PPIC
SP100
LAMB1
CFI
FAM114A1
MYH9
GRN
P4HB
WIPI1
RHOC
COL4A2
NMI
COL4A1
PDLIM1
IGFBP7
PLK3
PLSCR1
S100A6
CTSZ

TMEM147
MR1
CALU
IFITM2
F11R
CD40
CNPY4
MGAT1
TTC26
CDC42
CNIH4
ALG14
PLIN2
LMAN2
C2
SLC25A24
LATS2
TNFRSF14
ANO6
LYZ
CMTM6
ETHE1
LRRC25
CYBA
PLOD2
TMEM106C
S1PR3
MED8
TMEM165
TES
FCGRT
TRIP10
TEAD4
TCF7
CLIC4
MAGOH
FLNA
CALR
EFEMP2
CAPNS1
TMSB4X
ITPKC
PLEKHA4
LGALS1
GNS
PRSS23
IGFBP2
HK3
LATS2
EMP1
SEC24D
RELB
C1RL
DCBLD2
LOXL2
LY96
LUM
GLA
ARHGAP18
SERPINE1
REXO2
ARPC1B
ANXA5
RDH10
CAPZA1
RAB32
PLXND1
METTL7B
IFNGR2
PLBD1
ITGB3
PDIA4
SOCS3
LAMC1
CD63
SERPINH1
COL6A2
VIM
CARD16
COL3A1

RPN2
TWF2
FADD
PLAT
FN1
SLAMF8
TMBIM1
HK3
NOP10
TMEM194B
RGS19
SIL1
GRN
COL1A2
SRPR
CD248
FHOD1
ADAM12
NUP37
TCTN1
TRIM38
CD163
TMEM154
CCR5
DYRK3
TMEM149
LRRN4CL
VASN
OSMR
TP53I13
ARHGAP18
CCL5
SIGLEC9
HLA-A
IKBIP
PTX3
TNC
FES
RELL1
FAM115C

MRC2
SP140L
PDIA3
COL5A2
COL1A2
VASP
RIPK1
MYO1G
MMP14
PLP2
TUBB6
COL1A1
TGFBI
TMED9
HSPA5
OSMR
NAMPT
EMILIN1
TNFRSF1A
C1R
HEXB
NNMT
FAM20C
DPYD
CHI3L1
IFI30
MSR1
ADAM12
S100A11
MSN
SRPX2
ITGA5
FSTL1
TIMP1
FAM129A
EMP3
SYNPO
MYL12A
CCDC109B
GPX8

TMEM214
NEK6
SLC7A7
UNC93B1
LAMC1
ETV6
PPCS
IL2RG
ZNF600
C2orf28
DCTD
DOK3
ERI1
LGALS3
PPM1M
MRPS15
LOX
ITGB3
SLC2A4RG
LSM10
HLA-DRB1
DERL2
HSPB1
RBM47
CTSB
LPAR6
FAM26F
SERPINB6
RRAS
RPN1
EMR2
TMEM109
CAST
FUCA1
C9orf21
IQGAP1
PTRF
TUBA1C
TNFRSF12A
ANXA1
CLIC1

CA3

COL4A1

PVRL2

FNDC3B

MYD88

KCNE3

GALM

CDCP1

KDELR3

B3GNT5

ACTN1

NUCB1

MYOF

P4HB

ACOT9

C20orf30

WIPI1

TXNDC12

ENG

GMFG

FTL

CD164

PDLIM7

TPM4

HLA-DPB1

TMSB10

IGFBP2

SHISA5

FAM114A1

RELB

IFITM3

TRAM1

FAM46A

CLEC2B

COL4A2

PDIA5

NAGA

HLA-B

PECAM1

GBP2

CLEC18B

ZCCHC9

PRSS23

COL1A1

OLFML3

AIM1

GSDMD

LAP3

LEPRE1

LOXL2

ITGB1

SECTM1

NUAK2

BCL10

FCGBP

NCF1

CTBS

LOC100270710

DIRAS3

HLA-DMB

CKLF

CTSZ

VKORC1

SCPEP1

HLA-DPA1

LOC151534

SLC11A1

ARHGDIB

PDIA4

RAB42

SERPINE1

NCRNA00152

RIPK1

FXYD5

SLC35A2

GLB1

CMTM3

PDIA3P

FBP1

TPST1

IL10RB

PSMC2

C1orf54

RAB34

PARP9

PI4K2B

NTAN1

SLC30A7

APOBEC3F

SH3BGRL3

GADD45A

NAMPT

HMOX1

PIGT

DDB2

MS4A6A

MAP2K3

ZYX

ARPC1B

LEPREL1

SAT1

LY96

APOBEC3C

SERTAD1

SERPINB8

KDELR2

REEP4

MTMR11

OST4

SERTAD3

EMILIN1

C21orf63

TRIP6

ACTB

HSPA6

MGST2

HLA-DMA

SPATS2L

CTSA

VAMP8

ITPKC

SLC43A3

TCIRG1

SERPINB1

SHC1

STK40

ZBTB42

B2M

CNN3

RNASE4

NMI

BCL3

ARPC5

EMILIN2

HEBP2

ADAMTSL4

FCER1G

MYO1G

COL6A2

CD74

42262

GUSB

BST1

PRICKLE3

ORMDL2

SOD2

LOC541471

ALOX5AP

CTSC

AGTRAP

GPX7

RUNX1

ICAM1

ELF4

LGALS1

IRF1

CASP1

EMP1

GLT25D1

CD151

SOCS3

FCGR3A

TMED9

ISG20

SDF4

PLEKHA4

SP140L

MYL6

CD276

BACE2

ADPGK

GSTK1

HSD3B7

FUCA2

FOSL1

PQLC3

C5orf62

FAM20C

RAB27A

TMEM71

C1orf85

PDLIM1

STEAP3

GBP5

CAPZA1

MRC2

PCOLCE

CSDA

IQGAP1

RDH10

PFN1

PTRF

DDOST

DPYD

LTBR

C1S

MMP14

PLOD1

HEXB

RBMS1

FAM129A

FHL3

FAM70B

HLA-DRA

FAM176B

RAP1B

GLA

SLC16A3

GPR65

FAM109B

LSP1

MYCBP

FCGR2A

C21orf7

APOBEC3G

CHI3L1

SLC10A3

DAP

GBP1

AK2

NPC2

OSTC

CAPG

S100A10

GPX8

RNF135

MSR1

CD44

RAC2

CISH

VAMP5

WDR1

CASP6

IFNGR2

ANXA5

SP100

SERPING1

GNG12

ADPRH

SERPINA1

CARD16

KDELR1

TYMP

PDPN

TGIF1

PLK3

REXO2

CASP8

TUBB6

METTL7B

PTPN7

EDEM2

NNMT

IGFBP7

CD63

ZDHHC12

TUBA1C

BCL2L12

CFI

GNG5

MBD2

SYDE1

CCDC109B

RHOC

PLSCR1

C1RL

SPOCD1

PLP2

KIAA0040

UPP1

TMSL3

MSN

PLBD1

RAB32

VASP

FSTL1

SQRDL

ITGA5

SERPINH1

TNFAIP8

TAGLN2

SERPINA3

CD58

SLC39A1

S100A6

CHI3L2

PYGL

IFI30

PLAU

PLAUR

TNFRSF1A

C1R

TIMP1

MYL12A

EMP3

VIM

CASP4

CLCF1

S100A4

ANXA1

S100A11

CLIC1

TNFRSF12A

ANXA2P2
