# Supplementary material for: ANXA2 is correlated with the molecular features and clinical prognosis of glioma, and acts as a potential marker of immunosuppression
Source: Sci Rep. 2021 Oct 21;11:20839. doi: 10.1038/s41598-021-00366-8 (PMC8531374; doi:10.1038/s41598-021-00366-8)
Supplement: Supplementary file 2 — Supplementary Information 2. [file 41598_2021_366_MOESM2_ESM.pdf]

ANXA2 is correlated with the molecular features and clinical prognosis of glioma, and acts as a potential marker of immunosuppression

Kaiming Ma[1,2], Xin Chen[1,2], Weihai Liu[1,2], Yang Yang[1,2], Suhua Chen[1,2], Jianjun Sun[1,2], Changcheng Ma[1,2], Tao Wang[1,2], Jun Yang[1,2*]

[1] Department of Neurosurgery, Peking University Third Hospital, Beijing, China.

[2] Center for Precision Neurosurgery and Oncology of Peking University Health Science Center, Beijing, China.

*** Correspondence:** Jun Yang

Address: Department of Neurosurgery, Peking University Third Hospital, 49 North Garden Rd, Haidian District, 100191, Beijing, China.

Email： bysysjwk@126.com

Table S2. Immune-related genes significantly related to ANXA2 in TCGA database and in CGGA database.

Genes in TCGA database	Genes in CGGA database
ACTN1	COL1A1
ACTR3	COL1A2
ALOX5	COL3A1
ANO6	CYBA
ANXA1	FTL
APOBEC3C	FUCA1

APOBEC3F
APOBEC3G
APOL1
ARID5A
ARPC1B
ARPC2
ARPC5
ATP6V0A1
ATP8A1
B2M
BAK1
BATF3
BAX
BCL10
BCL3
BRI3
BST1
BST2
BTN2A2
BTRC
C1QA
C1QB
C1QC
C1RL
C1R
C1S
C2
C5AR1
CALR
CAP1
CAPZA1
CASP1
CASP4
CASP8
CAV1
CCL2
CCL5
CCR5
CCRL2
CD14

GLA
GUSB
CFH
ICAM1
CFI
IL2RG
ITGB3
LYZ
PROS1
THBD
PECAM1
HEXA
HEXB
SLC11A1
IGFBP2
SERPINE1
IL10RB
TGFB1
ANXA1
CHRNB2
IL1R1
ITGA4
PPIB
PTGER4
APOBEC3F
CXCR4
TNK2
CD276
CD74
CD151
TNFRSF1B
SP100
SERPINA3
ACTB
ACTN1
FLNA
CTSC
FCGRT
HSD3B7
CD58

CD151	DOK3
CD164	ANPEP
CD248	OSMR
CD276	CAV1
CD300A	BAK1
CD300LF	THRA
CD40	LY96
CD44	SIGLEC9
CD58	HLA-A
CD63	HLA-DQB1
CD68	CD300A
CD74	CD63
CD93	PSMD9
CDC42	CHI3L1
CFB	CRIP1
CFH	CTSZ
CFI	FABP5
CHI3L1	B4GALT1
CHRNB2	IL13RA1
CIITA	AHR
CKLF	CFB
CLCF1	C1R
CLEC2B	C1S
CLEC7A	C5AR1
CMTM6	CTSB
CMTM7	CTSD
COL1A1	DPP4
COL1A2	ETV6
COL3A1	GBP1
COTL1	GNS
CRIP1	GRN
CSTB	HK3
CTSA	HLA-DPB1
CTSB	HMOX1
CTSC	HSPA6
CTSS	ICAM3
CTSZ	IL7R
CXCR4	IRF1
CYBA	ITGA5
CYFIP2	LAIR1

DDOST	LGALS1
DOK3	MSN
DTX3L	MYH9
DYRK3	PSMB9
ELF4	PSMC2
EMILIN1	PSME2
ERP44	PTX3
ETV6	PYGL
F11R	RAC2
FADD	RAP2B
FASN	CCL2
FCER1G	SECTM1
FCGR2A	TRIM21
FCGR3A	THBS1
FCGRT	TLR2
FES	VIM
FLNA	GPR65
FN1	VAMP8
FTL	RIPK1
FUCA1	ADAM9
FUCA2	TNFRSF14
GBP1	FADD
GBP2	TNFRSF10D
GBP5	IQGAP1
GFRA1	BCL10
GLA	SOCS3
GLB1	B2M
GMFG	FCER1G
GNS	GBP2
GPNMB	PSMB8
GPR65	BST2
GRN	CALR
GSDMD	EPHA2
GUSB	NMI
HDAC1	SYNGR1
HDAC4	GMFG
HEBP2	SEC22B
HEXA	HDAC1
HEXB	MMP14
HK3	WDR1

HLA-A	CLEC2B
HLA-B	ALDOC
HLA-C	BCL3
HLA-DMA	DDOST
HLA-DMB	PDIA3
HLA-DOA	HCLS1
HLA-DPA1	PRKCE
HLA-DPB1	SH2B3
HLA-DQA1	HLA-B
HLA-DRA	IFNGR2
HLA-DRB1	LCP2
HMOX1	S100A11
HSD3B7	ARPC5
HSPA6	ARPC1B
ICAM1	ZMPSTE24
IFI30	TCIRG1
IFI44	HDAC4
IFITM2	NOD1
IFITM3	MYL9
IFNGR2	HLA-DMA
IGFBP2	CAPZA1
IL10RB	DNAJC3
IL13RA1	RRAS
IL2RG	IFI30
IL4I1	TRIM38
IQGAP1	PRDX4
IQGAP2	NPC2
IRAK4	IFITM2
IRF1	RELB
ISG20	FUT9
ITGA5	RAB32
ITGB1	GLIPR1
ITGB3	SPTBN2
KIF3A	EMILIN1
KIF3C	KIF3A
LAIR1	CD93
LAMP3	HEBP2
LAPTM5	APOBEC3C
LCP2	TMOD3
LGALS1	TBKBP1

LGALS3
LOXL3
LOX
LTBR
LY96
LYN
LYZ
MAPT
MLKL
MMP14
MR1
MSN
MUC1
MUL1
MYD88
MYO1C
MYO1G
NCAM1
NCF1
NME2
NMI
NOD1
NPC2
OSCAR
OSMR
PARP9
PDPN
PECAM1
PIK3R1
PLA2G6
PLAUR
PLAU
PLCB1
PLSCR1
PPIB
PRKCE
PROS1
PSMA5
PSMA7
SEC24D
SWAP70
RFTN1
C1RL
F11R
APLN
CMTM6
XKR8
SMPD3
HLA-DRA
SLAMF8
CD248
TWSG1
IFITM3
PLSCR1
COTL1
APOBEC3G
HLX
TMBIM1
MUL1
ZC3H12A
PDCD1LG2
FUCA2
CYSTM1
LOXL3
MYO1G
FN1
DTX3L
NFAM1
TXLNA
ITGA1
PHLPP1
ARID5A

PSMB8

PSMB9

PSMC2

PSMC4

PSMD9

PTGER4

PTPN2

PTX3

PYCARD

PYGL

RAB27A

RAB32

RAB34

RAC2

RAP1B

RAP2B

RBM47

RELB

RHOH

RIPK1

RNF135

RRAS

RUNX1

RUNX3

S100A11

S100A9

SASH3

SEC24D

SECTM1

SERPINA1

SERPINA3

SERPINB1

SERPINB6

SERPINE1

SERPING1

SHC1

SIGLEC9

SLAMF8

SLC11A1

SLC16A3

SLC7A7

SMPD3

SOCS1

SOCS3

SP100

SPTBN2

SWAP70

SYNGR1

TBC1D10C

TCF7

TCIRG1

TGFB1

TGFB2

TIFA

TMBIM1

TMEM176B

TMEM179B

TNFRSF14

TNK2

TNRC6C

TRIM21

TRIM23

TRIM38

TSC1

TTBK1

TTLL12

TWSG1

TXLNA

TXNDC5

TYROBP

UNC93B1

VAMP8

VIM

WDR1

XBP1

XKR8

ZFP36L2

ZMYND11

ZYX

ACTB
