# Supplementary material for: ANXA2 is correlated with the molecular features and clinical prognosis of glioma, and acts as a potential marker of immunosuppression
Source: Sci Rep. 2021 Oct 21;11:20839. doi: 10.1038/s41598-021-00366-8 (PMC8531374; doi:10.1038/s41598-021-00366-8)
Supplement: Supplementary file 3 — Supplementary Information 3. [file 41598_2021_366_MOESM3_ESM.pdf]

ANXA2 is correlated with the molecular features and clinical prognosis of glioma, and acts as a potential marker of immunosuppression

Kaiming Ma[1,2], Xin Chen[1,2], Weihai Liu[1,2], Yang Yang[1,2], Suhua Chen[1,2], Jianjun Sun[1,2], Changcheng Ma[1,2], Tao Wang[1,2], Jun Yang[1,2*]

[1] Department of Neurosurgery, Peking University Third Hospital, Beijing, China.

[2] Center for Precision Neurosurgery and Oncology of Peking University Health Science Center, Beijing, China.

*** Correspondence:** Jun Yang

Address: Department of Neurosurgery, Peking University Third Hospital, 49 North Garden Rd, Haidian District, 100191, Beijing, China.

Email： bysysjwk@126.com

Table S3. Inflammatory response-related genes and metagenes.

Genes	Metagenes
C1QB	HCK
C1QA	HCK
AIF1	HCK
LST1	HCK
DOCK2	HCK
LAPTM5	HCK

TYROBP	HCK
MS4A4A	HCK
MS4A6A	HCK
CD163	HCK
ITGB2	HCK
SLC7A7	HCK
LAIR1	HCK
HCK	HCK
TFEC	HCK
IFI30	HCK
MNDA	HCK
FCER1G	HCK
RNASE6	HCK
SLCO2B1	HCK
CCR1	HCK
IGSF8	IgG
ISLR2	IgG
IGSF21	IgG
IGSF1	IgG
IGSF22	IgG
IGDCC3	IgG
IGHD	IgG
IGSF11	IgG
IGSF5	IgG
IGSF6	IgG
IFIT1	Interferon
IFIT3	Interferon
IFI44L	Interferon
OAS3	Interferon
MX1	Interferon
RSAD2	Interferon
IFI44	Interferon
OAS2	Interferon
OAS1	Interferon
CD2	LCK
GZMK	LCK
GZMA	LCK
CD3D	LCK
CD53	LCK
LCK	LCK
ARHGAP15	LCK

CCL5	LCK
GMFG	LCK
SELL	LCK
STAT4	LCK
SAMSN1	LCK
RAC2	LCK
HCLS1	LCK
CCR7	LCK
PIK3CD	LCK
CORO1A	LCK
CD48	LCK
IL2RG	LCK
SH2D1A	LCK
SLAMF1	LCK
IL7R	LCK
INPP5D	LCK
KLRK1	LCK
FGL2	LCK
IRF8	LCK
SELPLG	LCK
IL10RA	LCK
SLA	LCK
CCR2	LCK
CSF2RB	LCK
HLA-E	MHC_I
HLA-H	MHC_I
HLA-B	MHC_I
HLA-J	MHC_I
HLA-F	MHC_I
HLA-G	MHC_I
HLA-A	MHC_I
HLA-C	MHC_I
HLA-L	MHC_I
HLA-DRB1	MHC_II
HLA-DRB5	MHC_II
HLA-DRB3	MHC_II
HLA-DPA1	MHC_II
HLA-DRA	MHC_II
HLA-DQA1	MHC_II
HLA-DQA2	MHC_II
HLA-DMA	MHC_II

HLA-DOA	MHC_II
HLA-DRB4	MHC_II
HLA-DMB	MHC_II
HLA-DQB1	MHC_II
HLA-DPB1	MHC_II
HLA-DQB2	MHC_II
CD74	MHC_II
PTPRC	MHC_II
HLA-DOB	MHC_II
HLA-DPB2	MHC_II
TAP1	STAT1
STAT1	STAT1
CXCL10	STAT1
CXCL11	STAT1
GBP1	STAT1
CXCL9	STAT1
