# Supplementary material for: ANXA2 is correlated with the molecular features and clinical prognosis of glioma, and acts as a potential marker of immunosuppression
Source: Sci Rep. 2021 Oct 21;11:20839. doi: 10.1038/s41598-021-00366-8 (PMC8531374; doi:10.1038/s41598-021-00366-8)
Supplement: Supplementary file 4 — Supplementary Information 4. [file 41598_2021_366_MOESM4_ESM.pdf]

ANXA2 is correlated with the molecular features and clinical prognosis of glioma, and acts as a potential marker of immunosuppression

Kaiming Ma[1,2], Xin Chen[1,2], Weihai Liu[1,2], Yang Yang[1,2], Suhua Chen[1,2], Jianjun Sun[1,2], Changcheng Ma[1,2], Tao Wang[1,2], Jun Yang[1,2*]

[1] Department of Neurosurgery, Peking University Third Hospital, Beijing, China.

[2] Center for Precision Neurosurgery and Oncology of Peking University Health Science Center, Beijing, China.

*** Correspondence:** Jun Yang

Address: Department of Neurosurgery, Peking University Third Hospital, 49 North Garden Rd, Haidian District, 100191, Beijing, China.

Email： bysysjwk@126.com

Table S4. Detailed information about the immune cell specific marker genes.

Immune cells	Marker genes
Macrophages	CD14
Macrophages	HLADRA
Macrophages	CD312
Macrophages	CD115
Macrophages	CD163
Macrophages	CD204

Macrophages	CD301
Macrophages	CD206
Neutrophils	CD11b
Neutrophils	CD16
Neutrophils	CD66b
Neutrophils	ELANE
MDSCs	CD14
MDSCs	CD16
MDSCs	CD33
MDSCs	ARG1
CD8T	CD3E
CD8T	CD8A
Tregs	CD3E
Tregs	CD4
Tregs	CD25
Tregs	FOXP3
CD4T	CD3E
CD4T	CD4
